# Median effective volume of 0.2% ropivacaine for ultrasound-guided supraclavicular brachial plexus block in children aged 1–6 years: a prospective dose-finding study

**DOI:** 10.3389/fped.2023.1157447

**Published:** 2023-05-12

**Authors:** Ling Liu, Fei Yang, Wen Gao, Shangyingying Li, Yaqiong Tian, Li Yang, Shengfen Tu

**Affiliations:** ^1^ Department of Anesthesiology Children’s Hospital of Chongqing Medical University, National Clinical Research Center for Child Health and Disorders, Ministry of Education Key Laboratory of Child Development and Disorders, Chongqing, China; ^2^ Chongqing Key Laboratory of Pediatrics, Chongqing, China

**Keywords:** median effective volume, ultrasound-guided, supraclavicular brachial plexus block, paediatrics, ropivacaine

## Abstract

**Objective:**

To determine the median effective volume (EV_50_) of 0.2% ropivacaine for ultrasound-guided supraclavicular brachial plexus block (SC-BPB) in children aged 1–6 years.

**Methods:**

Children aged from 1 to 6 years with an American Society of Anaesthesiologists (ASA) physical status I–II who were scheduled for unilateral upper extremity surgery at the Children's Hospital of Chongqing Medical University were recruited. All patients underwent surgery under general anaesthesia combined with brachial plexus block. SC-BPB was guided by ultrasound after anaesthesia induction, and 0.2% ropivacaine was given after localization. In the study, we used Dixon's up-and-down approach with an initial dose of 0.50 ml/kg. Considering the effect of the previous block, a successful or failed block could produce a 0.05 ml/kg decrement or increment in volume, correspondingly. The experiment was stopped when there were 7 inflection points. Using isotonic regression and bootstrapping algorithms, the EV_50_, the 95% effective volume (EV_95_) and the 95% confidence interval (CI) were calculated. The patients’ general information, postoperative pain scores, and adverse events were also recorded.

**Results:**

Twenty-seven patients were involved in this study. The EV_50_ of 0.2% ropivacaine was 0.150 ml/kg (95% CI, 0.131–0.169 ml/kg) and the EV_95_ (secondary metric) was 0.195 ml/kg (95% CI, 0.188–0.197 ml/kg). No adverse events occurred during the research study.

**Conclusions:**

For ultrasound-guided SC-BPB in children aged 1–6 years undergoing unilateral upper extremity surgery, the EV_50_ of 0.2% ropivacaine was 0.150 ml/kg (95% CI, 0.131–0.169 ml/kg).

## 1. Introduction

Brachial plexus blocks in children can be performed in all upper extremity surgical procedures (1). The supraclavicular approach is applicable for surgical analgesia in various parts of the upper extremity, including the arm, forearm, and hand. For this reason, SC-BPB has been referred to as the “spine of the upper extremity” (2). With the development of ultrasound technology, visualization has encouraged an increasing number of anaesthesiologists to increase their use of this method in paediatric patients (3). Ultrasound guidance has shown unparalleled advantages compared to previous peripheral nerve block techniques, such as the use of anatomical landmarks, induced paresthesia, and nerve stimulation (1, 4).

Ropivacaine is a long-acting amide local anaesthetic that has the added benefit of being less cardiotoxic than other local anaesthetics (5). This feature makes it a safer option. European and American recommendations recommend the use of ropivacaine for ultrasound-guided peripheral nerve block of the upper extremity in children (6).

Currently, dose studies on ropivacaine for ultrasound-guided brachial plexus block are mostly focused on adults (7–9) and other approaches of brachial plexus block in children (10). We have not found any published dose studies on upper extremity surgery under SC-BPB in children. The primary objective of this dose-finding study was to determine the EV_50_ of 0.2% ropivacaine for ultrasound-guided SC-BPB in children aged 1–6 years, that is, the dose that would induce successful surgical anaesthesia in half of the paediatric patients. We also calculated the dose required for an effective blockade in 95% of patients, which was our secondary metric. We hope that the results of our study can be used as a reference for clinical drug use.

## 2. Methods

### 2.1. Ethics

The study was approved by the Institutional Review Board of Children's Hospital of Chongqing Medical University, Chongqing City, China (Chairperson Professor: Zhongyi Lu, Approval number: 161-1/2020, Approval date: 12/23/2021). The trial was registered at the Chinese Clinical Trial Registry (Registration Number: ChiCTR2200057830, Registration Date: 18/03/2022).

After written informed consent was obtained, we prospectively recruited patients undergoing unilateral upper extremity surgery in March 2022 at Children's Hospital of Chongqing Medical University. Children aged 1–6 years with an ASA physical status I–II and a surgical site of the unilateral upper extremity were included. Patients were excluded if they had coagulopathy, were receiving anticoagulant therapy, had a puncture site infection, a known record of local anaesthetic hypersensitivity, nerve damage or upper extremity paresthesia, an intellectual disability, required bilateral surgery or refused to participate.

### 2.2. Study method

The dose, concentration, and volume of local anaesthetics are considered a “triangulated circular argument”. Therefore, when researchers conduct dose-finding studies using regional anaesthesia, one of the variables must be fixed in order to study the others (8). In this study, we selected a fixed concentration of 0.2% based on our clinical experience and previous research (10). According to the recommendation (6), a suitable dose of ropivacaine ranges from 0.5 to 1.5 mg/kg and can be selected for a successful and safe paediatric ultrasound-guided peripheral nerve block of the upper extremity. Our initial volume of 0.50 ml/kg was determined based on the median value of this dose and the gradient was fixed at 0.05 ml/kg. We used the Dixon “up-and-down” sequential allocation method in this study (11). Except for the first patient, the anaesthetic dose for each subsequent patient was determined based on the block effect of the previous patient. If the block failed, the next patient received an increment of 0.05 ml/kg on the failed dose. Conversely, a decrement of 0.05 ml/kg on the successful dose. When 7 inflection points were obtained (blocking effect went from a failure to a success), the study ended.

### 2.3. Anaesthesia procedures

Fasting for at least 6 h and abstaining from clear fluids for at least 2 h were the requirements for all children, and nurses arranged intravenous access in the ward in advance. After entering the operating room, the patient was continuously monitored for basic vital signs, including electrocardiogram (ECG), heart rate (HR), blood oxygen saturation (SPO2), respiratory rate (RR), body temperature (T), and mean arterial blood pressure (MAP). Anaesthesia was induced with midazolam 0.05 mg/kg, sufentanil 0.2 ug/kg, and propofol 3 mg/kg. During the operation, propofol 5 mg/kg h was used to maintain the depth of anaesthesia. All patients maintained spontaneous breathing, and oxygen masks were used to maintain oxygen supply.

### 2.4. Blocking process

**Figure 1:**
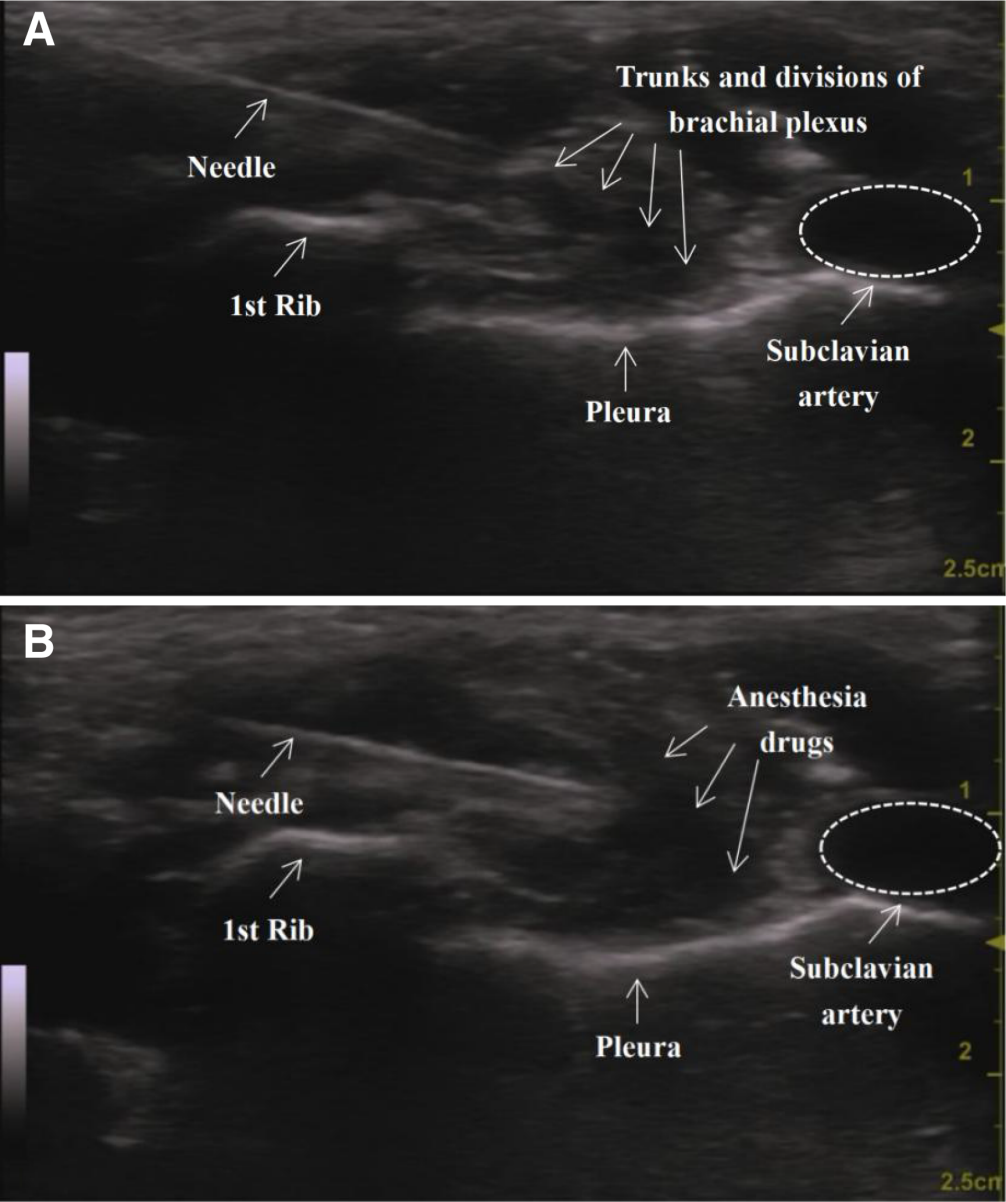
Ultrasound image of the supraclavicular approach before (**A**) and after (**B**) injection.

An ultrasound-guided SC-BPB was then performed. The patient lay flat on the bed with the arms in close contact with the sides of the body. The head was turned to the opposite side of the block to better expose the operating area. If necessary, access to the supraclavicular space could be improved by raising the ipsilateral shoulder to an appropriate height. After skin disinfection and draping, the ultrasound probe (GE venue50; GE, Boston, Massachusetts, USA) was wrapped in a sterile sleeve and placed in the supraclavicular fossa to locate the subclavian artery and the brachial plexus with a short axis. The brachial plexus and subclavian artery could be seen above the first rib. The subclavian artery is generally echoless, hypodense, pulsatile, and often appears in a round shape that is not easily flattened. We can also use colour Doppler to further confirm its identity. The brachial plexus trunk and branches appear as many hypoechoic honeycomb-like structures. The ribs are located on the medial and deep parts of the artery, with hyperechoic echo lines visible and shadows on the dorsal side. The pleura oscillates with breathing movements. Ultrasound image of the supraclavicular approach before (A) and after (B) injection is shown in Figure 1.

Then, an echogenic needle with a short-bevel tip (22 G, 50 mm, Stimuplex® D) was inserted from the side of the probe. After ensuring that the needle was fully visible, it could be slowly advanced by the in-plane technique until its tip was in a “corner pocket” (12) (defined as the junction of the first rib and the subclavian artery). A small amount of saline was used to confirm the location. Half of the anaesthetic was injected in this area after a bloodless draw. Later, the needle was positioned in the centre of the nerve cluster, and the remaining half of the drug was injected. Finally, a dynamic scan of the injection area could be performed to confirm the spread of the local anaesthetic. All operations were executed by the same experienced anaesthesiologist. Lung auscultation is necessary to detect the occurrence of pneumothorax in a timely manner. If suspected, a chest x-ray should be performed immediately.

### 2.5. Data acquisition

The HR and the MAP before skin laceration were taken as the baseline values. If the HR or the MAP after skin laceration was more than 20% above the value or the patient showed significant body movement, then propofol and sufentanil were administered to ensure that the patient could undergo the entire surgery in a painless state. The block was then defined as a failure. If the condition could not be improved or if the child had obvious respiratory suppression, a laryngeal mask or endotracheal tube was immediately placed to ensure ventilation. The block was defined as successful if the patient could undergo the entire procedure painlessly with stable vital signs.

After the surgery, the pumping propofol was stopped and the child was transferred to the postanesthesia care unit (PACU). An intravenous patient-controlled analgesia (PCA) device was given to the children after the operation. The patient could not be allowed to leave unless the Steward score was ≥4. The FLACC behavioural scale (13) for pain assessment was used for children in the PACU with postoperative pain that could not be accurately expressed. The main indicators include facial expression, legs, activity, cry, and consolability. The score is the sum of the above 5 indicators, with a minimum of 0 points and a maximum of 10 points (Table 1). The higher the score, the more noticeable the discomfort and pain. It was considered to be painless if the score was less than 4 points (10). For those with a score of ≥4, analgesic drugs could be administered and the block was then considered to be a failure. The patient was returned to the ward after becoming completely awake. Postoperative follow-up to monitor adverse reactions was conducted at a later time.

**Table 1:** FLACC behavioural scale.

Facial expression	No particular expression or smile;	0
	Occasional grimace or frown;	1
	Consistent grimace or frown, constant quivering chin, clenched jaw;	2
Legs	Normal position or relaxed;	0
	Uneasy, restless, tense;	1
	Kicking, or legs drawn up;	2
Activity	Lying quietly, normal position, moves easily;	0
	Squirming, shifting back and forth;	1
	Arched, rigid or jerking;	2
Cry	No cry or verbalization;	0
	Moans or whimpers, occasional complaint;	1
	Crying steadily, screams or sobs, frequent complaints;	2
Consolability	Content and relaxed;	0
	Reassured by occasional touching, hugging or being talked to, distractible;	1
	Difficult to console or comfort;	2

We recorded the general information of the patients, including name, sex, age, height, weight, ASA physical status, and surgical side. Meanwhile, the operation time, resuscitation time, and adverse events, including nausea, vomiting, bradycardia, hypotension, pruritus, respiratory depression and tracheal spasm were also documented.

### 2.6. Blinding method

The researchers conducting the study used a double-blind method. All SC-BPB procedures were performed by the same experienced physician who was not further involved in the study. Anaesthesia monitoring, data recording, and postoperative follow-up were performed by another study observer, whose duty was to assess the blocking effect and she was also unaware of the dosage. Medications prepared by the research assistant based on the evaluation results were given to another operational assistant. Only two assistants knew the volume of the anaesthetic. The operational assistant was only responsible for following the instructions of the doctor performing the nerve block to assist in injecting the anaesthetic, such as “inject half of the drug” or “inject the remaining half”. Meanwhile, the dose of ropivacaine was also unknown to the children and their guardians.

### 2.7. Statistical analysis

We applied the Dixon “up-and-down” sequential allocation method, which is a well-recognized, classic and widely used method for determining the half-effective dose of drugs in the field of anaesthesia.

The study was stopped after reaching 7 inflection points of the blocking effect from failure to success, and then EV_50_ and EV_95_ were statistically calculated by isotonic regression. For the 95% CI of the results, we used the bootstrap algorithm with 2,000 replicates. For the sake of higher accuracy, we used the estimate µ3 to represent the drug dose at which the target effect (0.5) was achieved. Statistical analysis was performed using the R4.2.0 software package (R Foundation for Statistical Computing, Vienna, Austria). Continuous variables are expressed as the mean ± standard deviation.

## 3. Results

**Figure 2:**
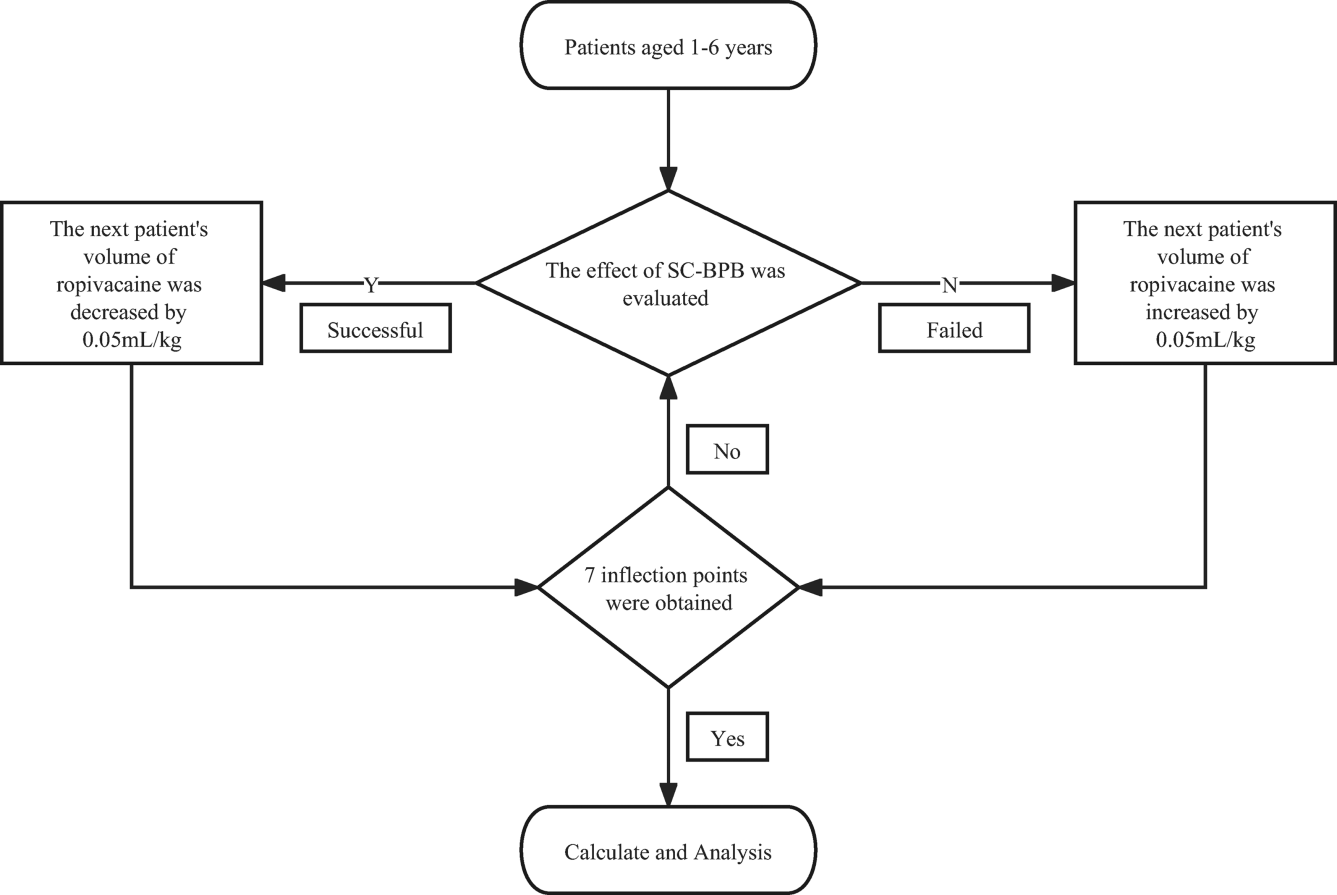
Flow diagram of the study.

A total of 31 children were recruited. Three were excluded for the following reasons: surgical protocol deviation (1 patient) and incomplete approval (2 patients). Twenty-eight children were allocated to the intervention. One patient was excluded due to preoperative analgesic use. Twenty-seven children were ultimately analysed. A flowchart of patient recruitment is shown in Figure 2.

The study on EV_50_ showed that seventeen children were successfully blocked, and ten children had failed blocks. Their demographic data are shown in Table 2. There was no significant difference in the general information of the patients between the failed and successful groups.

**Table 2:** Demographic data.

	Total (*n* = 27)	Success (*n* = 17)	Failure (*n* = 10)
Gender: male/female (*n*/*n*)	15/12	9/8	6/4
Weight (kg)	15.4 ± 4.6	14.8 ± 4.5	16.5 ± 4.9
Age (months)	40.0 ± 19.2	36.1 ± 18.8	46.7 ± 19.0
ASA physical status (Ⅰ/Ⅱ)	22/5	14/3	8/2
Operation side (L/R)	10/17	5/12	5/5
Operation time (min)	61.3 ± 26.7	60.8 ± 23.6	62.1 ± 32.6
PACU time (min)	40.4 ± 12.9	40.3 ± 14.0	40.5 ± 11.7

Data are presented as the mean ± SD.

**Figure 3:**
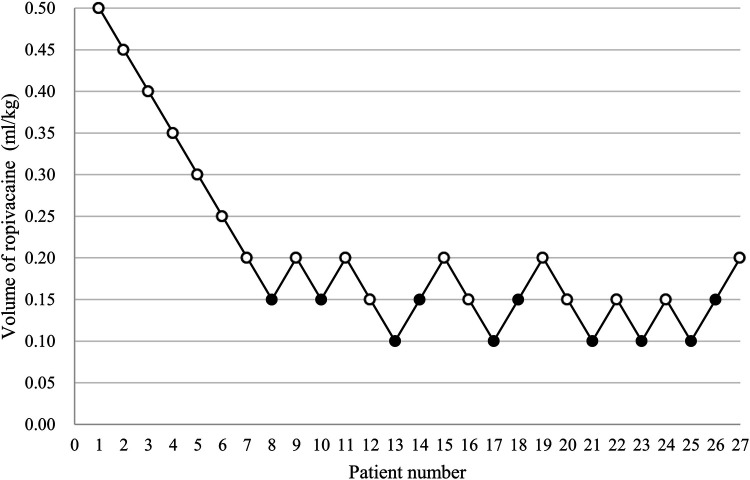
The patients’ block sequence diagram. (An open circle is used to represent the success volume; Failure volume is represented by a solid circle).

The patients' block sequence diagram is shown in Figure 3. The isotonic regression and bootstrapping algorithm were used to calculate EV_50_ and EV_95_. The EV_50_ of 0.2% ropivacaine was 0.150 ml/kg (95% CI, 0.131–0.169 ml/kg), and EV_95_ was 0.195 ml/kg (95% CI, 0.188–0.197 ml/kg).

## 4. Discussion

The concept of enhanced recovery after surgery (ERAS) refers to the adoption of a series of perioperative optimal measures with evidence-based medical science to reduce the physical and psychological traumatic stress of surgical patients and accelerate their recovery (14). With the increasing development of medical technology for paediatric surgery, ERAS has been implemented in the field of paediatrics, and its safety has been recognized by the majority of physicians (15). General anaesthesia combined with regional nerve block plays a key role in ERAS and has attracted clinical attention due to its advantages of reducing the dosage of general anesthesia, reducing stress response, and promoting rehabilitation. The core goal of ultrasound-guided nerve block anaesthesia is to deliver the optimal dose of local anaesthetic to the precise site to ensure optimal analgesia and minimal complications. However, currently, there is insufficient guidance on the dosage of ropivacaine in children, despite it being the most commonly used long-acting local anaesthetic of amides for nerve blockade. Our results for EV_50_ and EV_95_ can provide a basis for the accurate guidance of clinical medication and the formulation of related guidelines.

The SC-BPB method has been continuously improved. Researchers using the original approach suggest depositing the local anaesthetic into the visible nerve “clusters” dorsolateral to the artery (16). Subsequently, the proposal of a “corner pocket” was accepted as a new blocking method (17). Moreover, researchers began to explore the utility of injections in the “corner pocket” and the “clusters”, which showed evidence of a faster onset of action (18). In this study, we used the “dual injection” method (19), which is currently the most widely accepted among these methods.

In a randomized controlled trial involving 80 children aged 5–15 years, the supraclavicular approach was found to have a higher success rate than the infraclavicular approach because of its faster performance and easier implementation (20). The optimal dose of ropivacaine for SC-BPB under ultrasound guidance has been reported in the study (8), but it was performed in adults. Liang Chen et al. found that the minimum effective volume of 0.2% ropivacaine for ultrasound-guided brachial plexus block in preschool children was 0.185 ml/kg, but what they chose was the axillary approach (10). We speculate that their higher volume is due to anatomical factors. The supraclavicular approach has a dense plexus, while the axillary nerve distribution is scattered (21, 22).

Regarding the calculation of the sample size, it is necessary to make some clarifications. Studies with sequential designs must have a stopping rule. Our study was conducted until 7 crossover points (from failure to success of the block) were collected (11). The nonindependence and unknown distribution of data of the study prevent the development of theoretically rigorous rules to calculate the necessary sample size for a prespecified precision of the estimation of EV_50_ (23). Simulation studies suggest that including at least 20–40 patients will provide stable estimates of the target dose for most realistic scenarios (24). It is consistent with the point mentioned in the review (25).

It is worth mentioning that in several Dixon-based studies on drug dosage, the unit of drug gradient is usually defined in ml. Nevertheless, children's physical development varies greatly, and even at the same age, different children have different heights and weights. Therefore, in paediatric dosing studies, for the purpose of achieving individualized dosage, the gradient should be measured in ml/kg instead of in ml.

Among the failed cases, there were 4 under and 6 over 3 years old. From the numerical point of view, the older ones in the failed cases seem to be slightly more, but we think a small amount of data cannot easily conclude that the incidence of failure tends to increase with age. Of course, we do not rule out the correctness of this conclusion, but richer and deeper researches in the later stage need to be conducted to better demonstrate that in addition to the volume of anesthetic drugs, age may also be one of the reasons for failure.

Finally, this article also has some limitations. For children, it is necessary and ethical to relieve the pricking pain of the needle through an appropriate dose of analgesics. During the induction of anaesthesia, we gave each patient 0.2 ug/kg of sufentanil, a small dose that we do not rule out that may confound their response to surgical stimuli, but we think this effect was minimal. In addition, all children involved were aged 1–6 years. For those not in this age range, whether the volume of ropivacaine 0.2% is still appropriate remains to be further studied.

## Data Availability

The raw data supporting the conclusions of this article will be made available by the authors, without undue reservation.
